# Supplementary material for: Single-genome analysis reveals a heterogeneous association of the herpes simplex virus genome with H3K27me2 and the reader PHF20L1 following infection of human fibroblasts
Source: mBio. 2024 Feb 27;15(4):e03278-23. doi: 10.1128/mbio.03278-23 (PMC11005365; doi:10.1128/mbio.03278-23)
Supplement: Supplemental Figures — Figures S1-S3. [file mbio.03278-23-s0001.pdf]

Supplementary Information

Manuscript title: Single-genome analysis reveals heterogeneous association of the Herpes Simplex Virus genome with H3K27me2 and the reader PHF20L1 following infection of human fibroblasts.

Supplementary figure 1

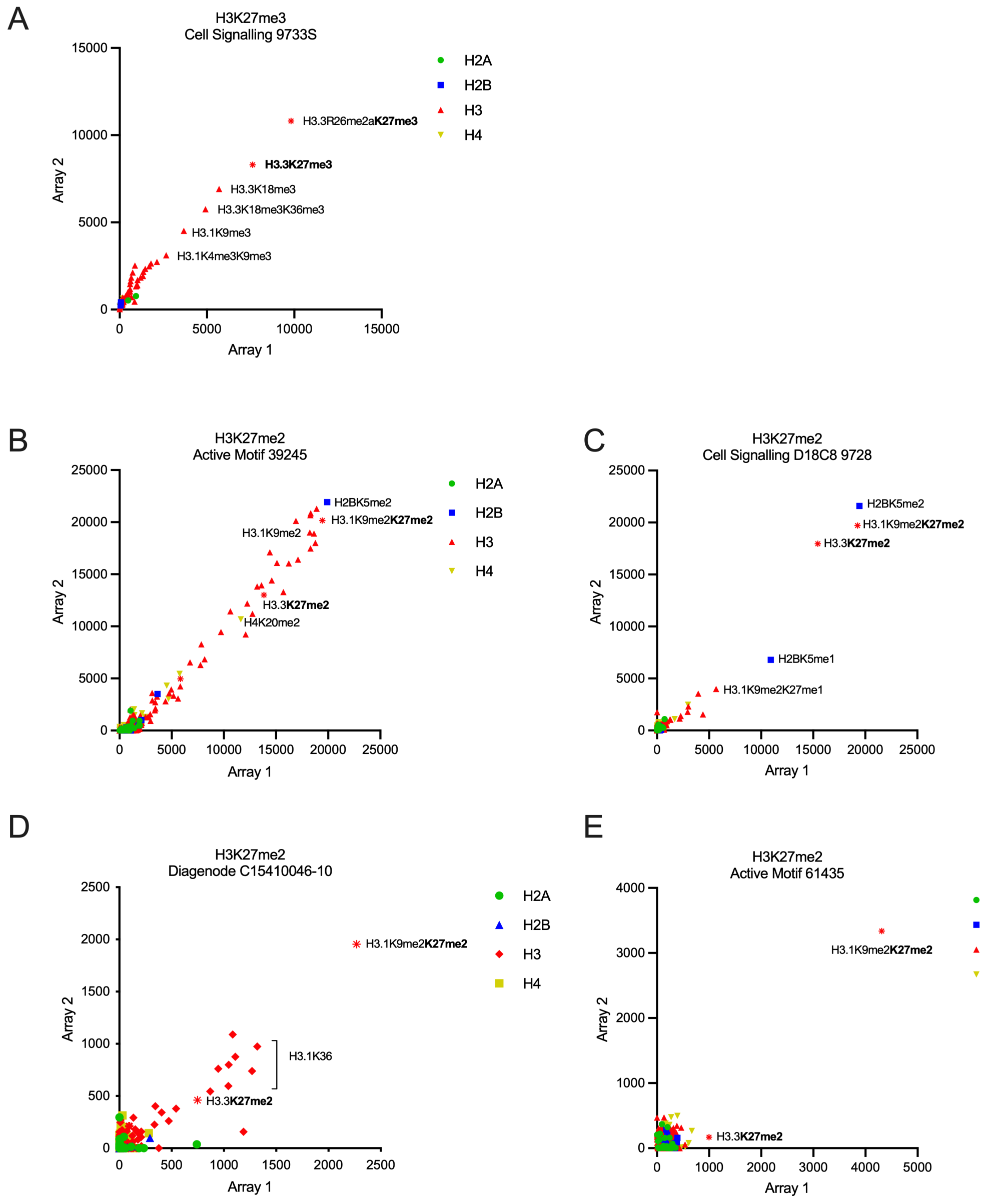

Figure S1. Histone peptide binding arrays for H3K27me3 and H3K27me2 antibodies show variable target specificity and non-specific binding affinities. Scatter plots of two binding array data sets from the same antibody sample, one dataset on each axis to indicate reproducibility. Depicted are the relative binding intensities for the chosen commercial antibodies. Labels are bolded where the antibody's target residue is included in a combination of histone peptide modifications. Other notable non-specific binding partners are also labeled.

Supplementary Figure 2

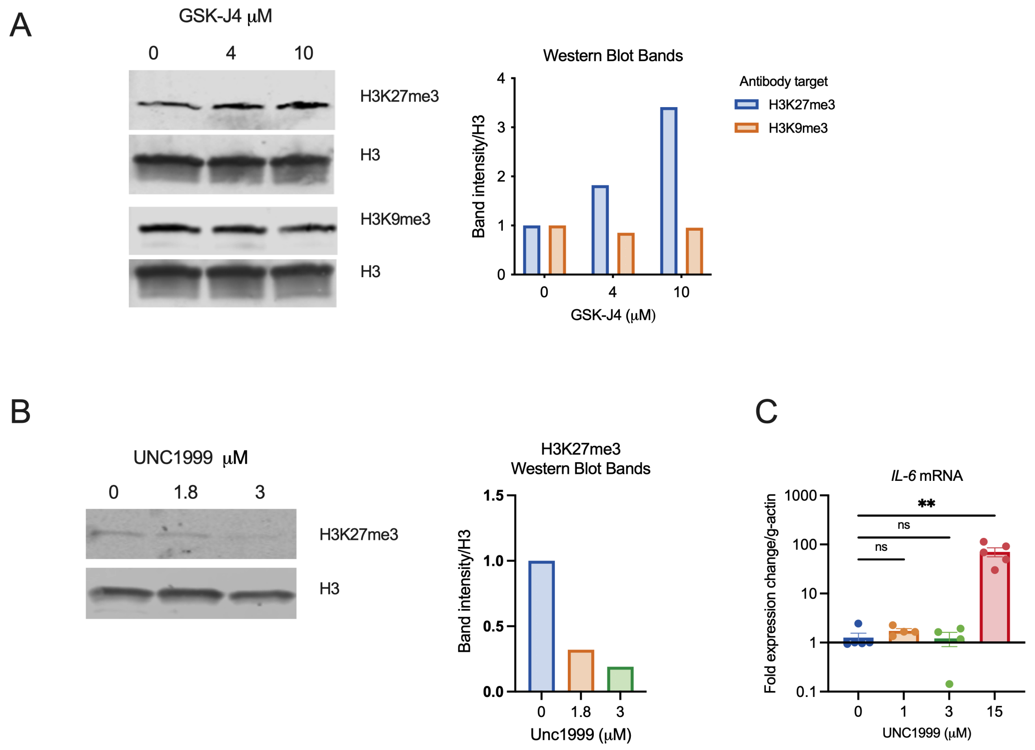

Figure S2. Validation of inhibitor activity and lack of interferon-stimulated gene induction. (A) HFFs were treated with UNC1999 at indicated concentrations for 4 days, with fresh inhibitor added once on day 2. The cumulative effect of UNC1999 on cellular chromatin was assessed from histone extracts blotted for H3K27me3. Li-Cor band quantification is normalized to total H3 bands, relative to untreated cells. (B) Cumulative effects of treatment with GSK-J4 for four days on cellular chromatin, assessed by blotting histone extracts for both H3K27me3 and H3K9me3. Li-Cor band quantification in A and B was normalized to total H3 bands, relative to untreated cells. (C) IL-6 expression measured by RT-qPCR of cDNA made from HFFs treated with indicated concentrations of UNC1999 for 5 hours.

Supplementary Figure 3.

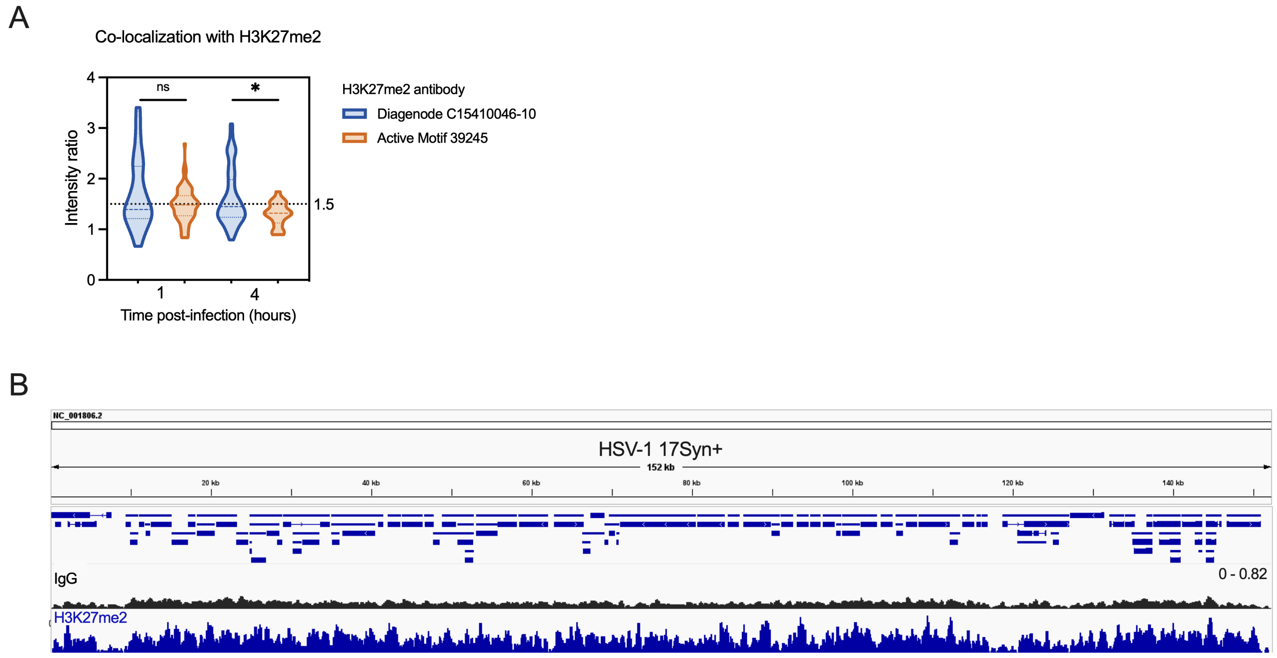

Figure S3. Certain H3K27me2 antibodies show evidence of non-specific binding to the viral genome. (A) Comparison of co-localization with H3K27me2 immunostained with two different antibodies at 4 hpi (Kolmogorov-Smirnov test.) Adjusted p-value *=<0.05. (B) 17Syn+ genome coverage from HFFs 1 hpi, from a single replicate of CUT&RUN with control IgG and H3K27me2 antibodies (Diagenode C15410046-10).
